# Territory location and quality, together with climate, affect the timing of breeding in the white-throated dipper

**DOI:** 10.1038/s41598-019-43792-5

**Published:** 2019-05-21

**Authors:** A. L. K. Nilsson, T. Slagsvold, O. W. Røstad, E. Knudsen, K. Jerstad, L. Cadahía, T. Reitan, M. Helberg, B. Walseng, N. C. Stenseth

**Affiliations:** 1 Centre for Ecological and Evolutionary Synthesis (CEES), Department of Biosciences, University of Oslo, P.O. Box 1066 Blindern, NO-0316 Oslo, Norway; 2 Faculty of Environmental Sciences and Natural Resource Management, Norwegian University of Life Sciences, P.O. Box 5003 NMBU, NO-1432 Ås, Norway; 3 Present Address: NLA University College Oslo, P.O. Box 7153 St. Olavs plass, NO-0130 Oslo, Norway; 4 Jerstad Viltforvaltning, Aurebekksveien 61, NO-4516 Mandal, Norway; 5 Østfold University College, P.O. Box 700, NO-1757 Halden, Norway; 6 Norwegian Institute for Nature Research, Gaustadalléen 21, NO-0349 Oslo, Norway

**Keywords:** Climate-change ecology, Phenology, Evolutionary ecology

## Abstract

Recent climate change has led to advanced spring phenology in many temperate regions. The phenological response to variation in the local environment, such as the habitat characteristics of the territories birds occupy, is less clear. The aim of this study is to understand how ecological conditions affect breeding time, and its consequences for reproduction, in a white-throated dipper *Cinclus cinclus* population in a river system in Norway during 34 years (1978–2011). Hatching date advanced almost nine days, indicating a response to higher temperatures and the advanced phenology in the area. Earlier breeding was found in warm springs and at lower altitudes. High population density facilitated earlier breeding close to the coast. Furthermore, when population density was low, breeding was early at territories that were rarely occupied, while in years with high density, breeding was early at territories that were frequently occupied. Also, when population density was low, earlier breeding occurred at territories that on average produced more offspring than other territories, while there was no difference in breeding time in high population years. Selection for early breeding was dependent on spring temperatures and high spring temperatures contributed to higher breeding success during the study period. We found that breeding phenology may have strong effects on fitness in the white-throated dipper, and thus that breeding time is an important ecological factor in a species that feeds mainly on aquatic rather than terrestrial prey.

## Introduction

Phenology is the study of the annual timing of recurring life history events, like the timing of migration and breeding in seasonal environments. Phenology has lately received large attention because phenological traits are responding to recent climate change, where the timing of spring events in particular has advanced. Among the studied taxa, birds show a particularly strong phenological response to climate change^1^. This response can be the result of the direct effects of a changing climate on the mechanisms regulating endogenous timing, e.g., physiology, or it can be caused by indirect effects through the food chain^2^. Most of the reported responses are due to phenotypic plasticity, with individuals adjusting their timing of breeding to annual variation in temperature^3^. For successful breeding, some passerine birds are dependent on food resources that are limited to a brief time window when they are abundant^4,5^, while others are not subject to such time constraints^6^. In this paper, we study the phenological response to environmental variation in the white-throated dipper *Cinclus cinclus* (hereafter dipper), where the time constraints for successful breeding potentially posed by the food resources are unknown. We address the impact of ecological factors on individuals at the scale at which they interact with their environment, namely the scale of the territories birds occupy. Habitat heterogeneity may make individuals in the same population experience different environments, and, thus, selection may favour sensitivity to local scale variation^7^. Therefore, we here study issues related to local climate, territory location and quality, and the role of intraspecific competition, in addition to spring temperatures measured at a regional level.

At northern latitudes, passerine egg-laying often occurs simultaneously with leafing of the trees and is strongly correlated with ambient temperatures^8^. However, differences in ambient temperatures can also be found at local scales, e.g., due to altitudinal gradients^9,10^. At increased elevation, the timing of egg-laying in great tits *Parus major* is delayed while clutch size and breeding success is reduced^11^, which might be explained by temperature and food availability^12^ through both proximate and ultimate mechanisms. Therefore, both latitude and altitude may play a role in determining phenology.

Habitat quality influences fitness, and some territories are therefore preferred to others^13^. For example, in a study of black kites *Milvus migrans*, high occupancy territories produce most of the young subsequently entering the breeding population, because these are often associated with lower predation risk and higher food abundances^14^. In general, older or dominant individuals often occupy high quality territories^15^. Moreover, birds settle earlier on high quality territories (especially migrants), suggesting that territory quality may be associated with the time of breeding.

Breeding population density is an indication of the level of intraspecific competition in a population and can hence influence the availability of food resources and nest sites^13^. Increased breeding population density might lead to a scenario where all individuals suffer, or to a scenario where high-quality territory holders suffer little, but more low-quality territories are occupied than when breeding population density is low^16^. In dippers, the location of the nest sites in some territories might be unfavourable despite an abundance of food and are therefore only occupied in high-density years. Breeding population density can therefore have a strong impact on average breeding performance^17^. An early start of breeding may be particularly beneficial under high population densities to utilize the resources as long as they last, thereby giving the offspring a good (and early) start in a competitive environment.

We used data from a long-term study on the dipper in southernmost Norway (1978–2011) to address how the local climate, territory location and quality, and intraspecific competition affect the timing of breeding. The dipper is a medium-sized single-brooded bird (50–70 g) breeding in mountainous regions across the Palearctic. The five dipper species in the world occupy a special feeding niche by preying upon aquatic invertebrates and fish in running freshwater, with breeding occurring in the immediate vicinity of fast-flowing rapids. The birds in our population are partial migrants, and in particular in cold winters when most of the river system freezes, the resident fraction of the population is apparently severely reduced^18^. Breeding phenology was estimated as the hatching date of the first clutch of a female. In summary, hatching date was estimated based on the growth trajectory of the largest nestling in each brood (see methods for further details). Thus, phenology of nests that did not produce young, or produced young that were not measured, could not be included in the analyses. We measured the quality of each territory as overall mean territory occupancy rate, overall mean territory success rate, and overall mean territory clutch and brood size. The territories are spatially fixed, due to the dippers’ special requirements for a nest site, and only a certain number of physical locations in the river system are suitable, which are occupied year after year (see methods for further details). As in the black kite, some territories are obviously preferred over others by the dippers in our study population. In addition, in American dippers *C. mexicanus*, fidelity to the breeding territory is very high, about 70% of both males and females return to the same territory^19^, which lends support to the view that the territory is an important breeding component requiring further study.

In this study, we explore (i) whether breeding time has advanced over the study period, and whether increased spring temperatures have led to the advancement. Then we investigate, in an explorative context, (ii) the causes of variation in the timing of breeding, namely: whether breeding was earlier in warm than in cold springs, whether breeding was earlier closer to the coast and at lower altitudes compared to inland and at higher altitudes, whether local habitat quality at the territory level affected the variation in breeding time, and whether intraspecific competition affected breeding time, where high population density, and thus more competition, might select for earlier breeding. Finally, we consider (iii) the consequences of variation in the timing of breeding, by studying the fitness consequences (i.e. the number of fledglings or recruits) of the phenological response to the environment, and whether this response differs between years and might have been caused by increasing spring temperatures.

## Results

### Temporal trends and climate effects

**Figure 1:**
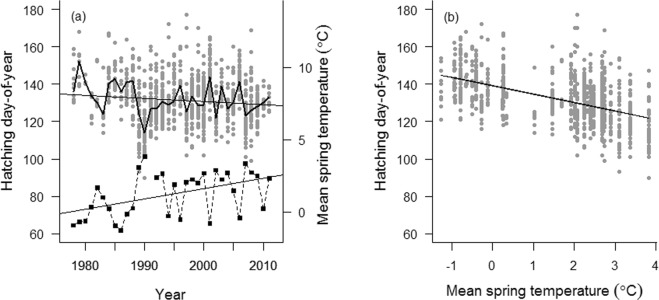
The annual variation in hatching date and spring temperatures. (**a**) Annual variation in hatching date (hatching day-of-year, solid line) and mean spring temperatures (Feb–Apr, broken line) in the white-throated dipper population in Lyngdalselva, and (**b**) the effect of mean spring temperature on annual hatching date in the study area (1978–2011). The trend line in each time series is denoted with a solid line. N = 1045.

The median date for initiation of breeding (hatching date of first nestling in a brood) during 1978–2011 was 10^th^ of May (SD = 13 days, IQR = 3^rd^–19^th^ of May). Over the length of the study period, the start of breeding advanced almost 9 days (Conditional R^2^ = 0.31, marginal R^2^ = 0.03; b = −0.26, t = −2.0, df = 35.5, P = 0.056, random effects: year SD = 6.8, residual SD = 10.5; Fig. 1a). Over the same period, mean spring temperature (Feb–Apr) increased by 2.5 °C (b = 0.07, t = −3.0, df = 31, R^2^ = 0.22, P = 0.006; Fig. 1a). Mean spring temperature accounted for much of the trend in breeding phenology (Conditional R^2^ = 0.30, marginal R^2^ = 0.23; year: b = 0.03, df = 26.2, t = 0.4, P = 0.7; spring temperature: b = −4.3, df = 21.5, t = −8.5, P < 0.0001, random effects: year SD = 3.3, residual SD = 10.5; Fig. 1b). When comparing a linear with a non-linear phenological response to mean spring temperature with a likelihood ratio test, the linear was preferred (generalised additive mixed model; nonlinear: edf = 1.5, F = 63.4, P < 0.0001; Likelihood ratio test: BIC(nonlinear) = 7627.0, BIC(linear; the null hypothesis) = 7620.4, likelihood ratio = 0.2, P = 0.6; Fig. 1b).

### Causes of variation in the timing of breeding

**Figure 2:**
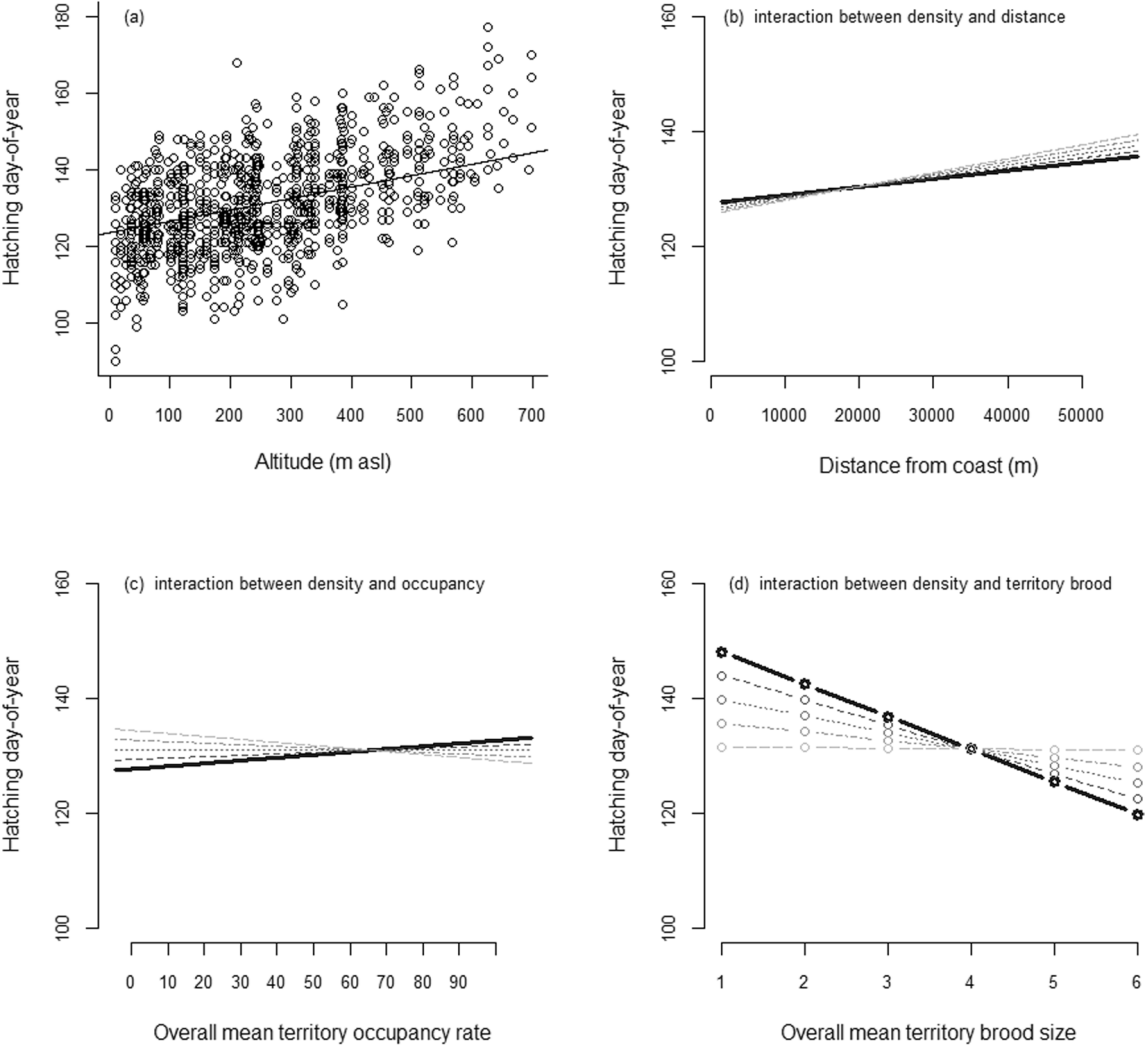
The causes of variation in the timing of breeding modelled in a linear mixed-effects model in the white-throated dipper in Lyngdalsvassdraget 1978–2011. Effects are shown for the following fixed effects, using model predictions and a restricted data set: (**a**) altitude (m above sea level), (**b**) the interaction between distance from the coast (m) and population density, where population density is ordered from low to high with decreasingly light grey tones, (**c**) the interaction between overall mean territory occupancy rate and population density, where population density is ordered as in (**b**), and (**d**) the interaction between overall mean territory brood size and population density, where population density is ordered as in (**b**) and (**c**),with the crossed random effects year, female identity and territory identity, on the hatching day-of-year. N = 992.

Mean spring temperature, altitude, distance from the coast, population density, overall mean territory occupancy rate, overall mean territory success rate, and overall mean territory clutch, brood size were included in a linear mixed model framework in order to explain the variation in hatching date using an explorative approach. Because we were interested in potential interactions with mean spring temperature and population density, we compared models with the additional two-way interactions between them and the other variables. We used model averaging to rank models with the Bayesian Information Criterion, BIC, with the random intercepts year, territory identity and female identity. The top model had considerably higher BIC weight (BIC_w_ = 0.71) than the lower ranked models (three models, ΔBIC > 4.0, BIC_w_ ≤ 0.09; Table 1). The top model included spring temperature, altitude, distance from the coast, population density, overall mean territory success rate and overall mean territory brood size, in addition to the interactions between density and distance, density and overall mean territory success rate, and density and overall mean territory brood size, respectively, (Table 2), and it explained 78% of the variation in hatching date (conditional R^2^ = 0.78, marginal R^2^ = 0.61; random effects: year SD = 3.4, territory id SD = 2.7, female id SD = 2.9, residual SD = 5.9). Hatching date advanced with increasing spring temperature, and with decreasing altitudes (Fig. 2a). Hatching date advanced with increasing population density at coastal territories, while it was delayed with increasing population densities inland (Fig. 2b). At low population density, hatching date at seldom occupied territories was earlier, while at high population density, often occupied territories hatched earlier (Fig. 2c). In contrast hatching was earlier at territories with large overall mean territory brood size during low population density years, while at high population density hatching occurred at the same time at all territories (Fig. 2d). Spring temperature (25.0%), the interaction between density and overall mean territory brood size (21.4%), altitude (13.7%) and density (12.0%) explained most of the variation, while overall mean territory brood size (7.8%), year (5.7%), female identity (4.1%), overall mean territory occupancy rate (2.6%), distance from the coast (1.8%), the interaction between density and distance from the coast (1.3%), and overall mean territory occupancy rate (1.1%) explained a smaller part of the variation in breeding time.

**Table 1:** The top ten models given by a model selection process where timing of breeding were explained by spring temperature (temp), altitude (alt), distance from the coast (dist), population density (dens), the territory quality variables overall mean territory occupancy rate (tocc), brood size (tbrood), and success rate (tsuccess), in addition to all interactions with spring temperatures, and all interactions with population density, in the white-throated dipper population in Lyngdalselva 1978–2011.

Models	df	BIC	ΔBIC_c_	BIC_w_
dist + tocc + dens + alt + temp + tbrood + dist × dens + tocc × dens + tbrood × dens	14	6789.2	0.0	0.71
dist + tocc + dens + alt + temp + tbrood + dist × dens + tocc × dens + tbrood × dens + alt × temp	15	6793.3	4.1	0.09
dist + tocc + dens + alt + temp + tbrood + tocc × dens + alt × dens + tbrood × dens + alt × temp	15	6793.6	4.4	0.08
dist + tocc + dens + alt + temp + tbrood + dist × temp + tocc × dens + tbrood × dens + alt × temp	15	6793.9	4.7	0.07
dist + tocc + dens + alt + temp + tbrood + dist × temp + tocc × dens + alt × dens + tbrood × dens + alt × temp	16	6796.9	7.7	0.02
dist + tocc + dens + alt + temp + tbrood + dist × dens + dist × temp + tocc × dens + tbrood × dens + alt × temp	16	6798.4	9.2	0.01
dist + tocc + dens + alt + temp + tbrood + dist × dens + tsuccess + tocc × dens + tbrood × dens + alt × temp	16	6798.8	9.6	0.01
dist + tocc + dens + alt + temp + tbrood + dist × temp + alt × dens + alt × dens + tbrood × dens + alt × temp	16	6798.9	9.7	0.01
dist + tocc + dens + alt + temp + tbrood + dist × dens + tocc × dens + tbrood × dens + alt × temp + tbrood × temp	16	6799.0	9.8	0.01
dist + tocc + dens + alt + temp + tbrood + dist × dens + tocc × dens + tbrood × dens + temp × dens + tbrood × dens + alt × temp	16	6799.0	9.8	0.01

**Table 2:** Timing of breeding explained by the model with the lowest BIC, consisting of spring temperature (temp), altitude (alt), distance from the coast (dist), population density (dens), the territory quality variables overall mean occupancy rate (tocc) and brood size (tbrood) in the white-throated dipper in Lyngdalselva 1978–2011.

Fixed effects	Estimate	t
intercept	152.00	24.1
temp	−4.76	−9.6
alt	0.03	8.4
dist	0.0001	1.9
dens	−0.18	−2.5
tocc	0.07	1.8
tbrood	−6.76	−4.5
dist x dens	0.000001	1.7
tocc x dens	−0.0010	−2.4
tbrood x dens	0.06	3.3

### Consequences of variation in the timing of breeding

**Figure 3:**
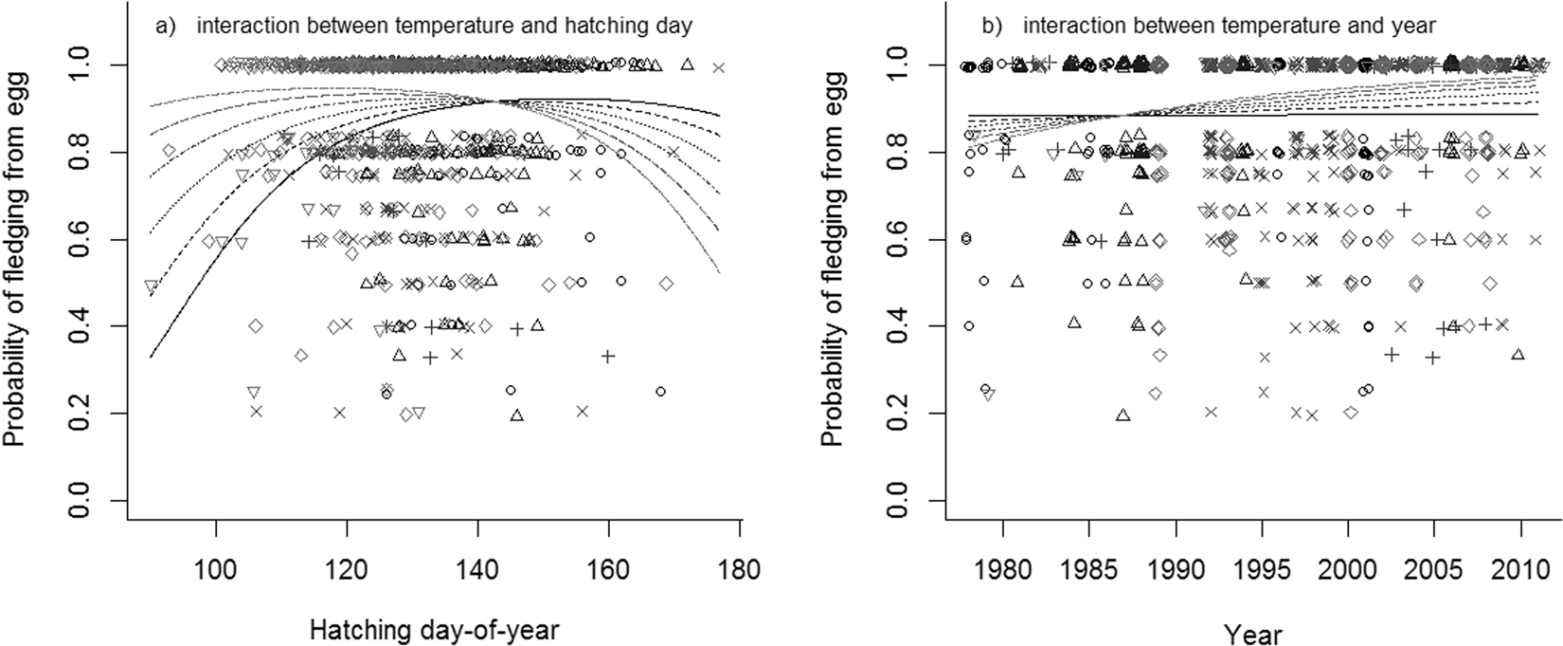
The consequences of variation in the timing of breeding, measured as the number of fledglings given the number of eggs laid in the white-throated dipper in Lyngdalsvassdraget 1978–2011. The effects are shown for the interactions (**a**) between spring temperature and hatching day-of-year, where spring temperature is ordered from low to high with increasingly light grey tones where −1.5–−0.5 is symbolised by solid line and circles, −0.5–0.5 °C by broken line and triangles, 0.5–1.5 °C by dotted line and plus symbols, 1.5–2.5 °C by broken-dotted line and crosses, 2.5–3.5 °C by long-stapled line and diamonds, and 3.5–4.5 °C by long-stapled-dotted line and downwards-facing grey triangles, and (**b**) between spring temperature and year, where spring temperature is ordered from the earliest to the latest as in (**a**), from a mixed-effects model including the most influential predictor variables (RI > 0.5) with the random crossed effects female and territory identity. N = 999.

In an explorative approach, we modelled the probability of fledging in a generalized linear mixed-effects model framework with the number of fledglings, given the number of eggs laid, as the response variable with the crossed (i.e. non-nested) random intercepts territory and female identity. The random intercept year had only a minor impact and it was consequently excluded from the models (including year: BIC = 1940.6, excluding year: BIC = 1933.7). Hatching date, year, spring temperature, population density and altitude, and the interactions between year and spring temperature, year and population density, spring temperature and population density, and altitude and population density, respectively, were included as predictor variables. We used model averaging to rank models based on BIC. The top model had considerably higher Bayesian weight (BIC_w_ = 0.74) than the lower ranked models (ΔBIC ≥ 4, BIC_w_ ≤ 0.08; Table 3) The top model included hatching date, the quadratic effect of hatching date, year, spring temperature, and the interactions between hatching date and temperature, and temperature and year, respectively (random effects: territory id SD = 0.4, female id SD = 0.8; Table 4). The probability of fledging had a later optimal hatching date at low temperatures than at high temperatures (Fig. 3a). The probability of fledging increased during the study period with higher spring temperatures, while low spring temperatures had little effect over time on the probability of fledging (Fig. 3b).

**Table 3:** Top five models for explaining breeding success, measured as the number of fledglings given the number of eggs, with hatching date (date), year, spring temperature (temp), population density (dens), and altitude (alt), in addition to all interactions with spring temperature and population density, respectively, in the white-throated dipper in Lyngdalselva 1978–2011.

Model	df	BIC	ΔBIC	BIC_w_
date + date^2^ + temp + year + date × temp + year × temp	9	1886.2	0.0	0.74
date + date^2^ + temp + year + dens + date × temp + year × dens	10	1890.7	4.4	0.08
date + date^2^ + year + dens + alt + date × dens + dens × alt	10	1890.9	4.6	0.07
date + year + dens + alt + date × dens + year × dens	10	1891.3	5.1	0.05
date + date^2^ + temp + year + alt + date × temp + year × temp	10	1891.4	5.2	0.05

**Table 4:** Breeding success, measured as the number of fledglings given the number of eggs, explained by the model with the lowest BIC, consisting of hatching date (date), spring temperature (temp), and year in the white-throated dipper in Lyngdalselva 1978–2011.

Fixed effects	Estimate	Z
intercept	−42.5	−2.0
date	0.2	2.6
date2	−0.0008	−2.5
year	0.01	1.4
temp	−23.8	−2.5
date × temp	−0.01	−2.7
year × temp	0.01	2.6

**Figure 4:**
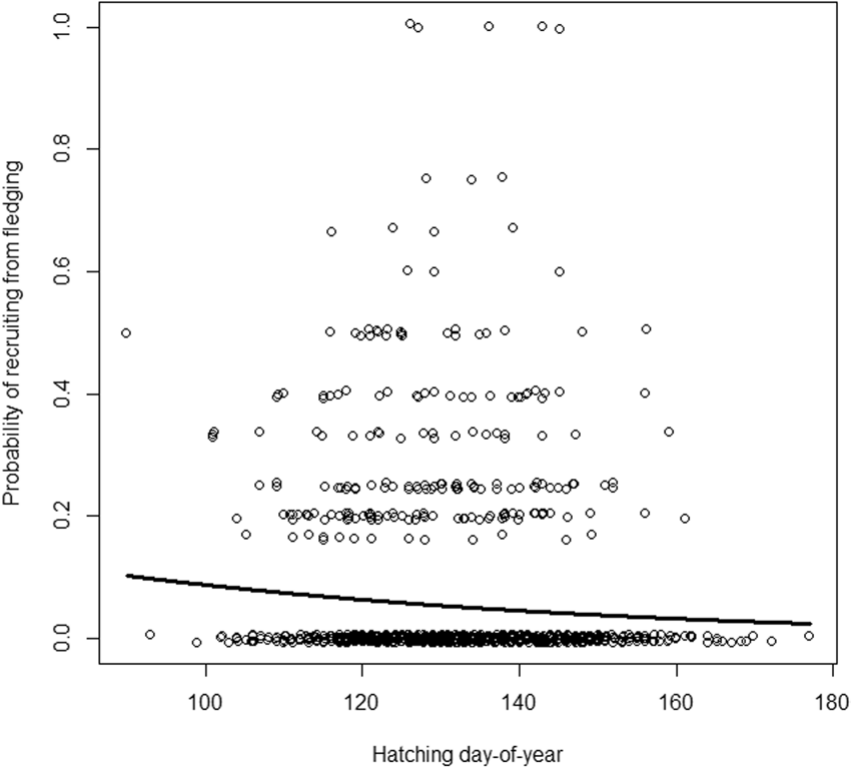
The consequences of variation in the timing of breeding, measured as the number of recruits given the number of fledglings, depending on the breeding time, measured as the hatching day-of-year, in the white-throated dipper in Lyngdalsvassdraget 1978–2011. N = 999.

Similarly, we modelled the probability of recruiting into the breeding population with the response variable the number of recruits, given the number of fledglings. We compared a range of likely models with the predictors hatching date, year, spring temperature, population density and altitude, and the interactions between year and spring temperature, year and population density, spring temperature and population density, and altitude and population density, respectively, using BIC. Female identity could be removed from the crossed random effects (including female id: BIC = 1385.3, excluding female id: BIC = 1379.4). The top model had considerably higher Bayesian weight (BIC_w_ = 0.70) than the lower ranked models (ΔBIC ≥ 4, BIC_w_ ≤ 0.20; Table 5), and included only hatching date (b = −0.02, z = −2.5; random effects: year SD = 0.6, territory id SD = 0.5; Fig. 4). In contrast to the results for the number of fledglings, given the number of eggs laid, spring temperature was not an influential predictor variable for the number of recruits, given the number of fledglings (Table 5). In conclusion, early breeding pairs produced more recruits than late breeders.

**Table 5:** Top five models explaining breeding success, measured as the number of recruits given the number of fledglings, with hatching date (date), year, altitude (alt), population density (dens), all interactions with spring temperature and interactions between population density, respectively, in the white-throated dipper population in Lyngdalselva 1978–2011.

Model	df	BIC	ΔBIC	BIC_w_
date	4	1316.5	0.0	0.70
Alt	4	1319.0	2.5	0.20
date + dens	5	1322.4	5.9	0.04
date + alt	5	1323.0	6.4	0.03
date + year	5	1323.0	6.5	0.03

## Discussion

In our study population, hatching date has advanced almost nine days during the 34 years of the study. Seemingly, the dipper has advanced its breeding time to adjust for the earlier spring, indicated by the increased mean spring temperatures. Spring temperatures, altitude, and the interactions between population density and overall mean territory brood size, population density and distance from the coast, and population density and overall mean territory occupancy rate, respectively, explained 78% of the variation in breeding time. In general, birds breeding early enjoyed fitness benefits, which to some extent were explained by the effect of increased spring temperatures on fledging success. Fledging success also increased over the study period in response to warm spring temperatures, whereas there was no temporal trend in cold springs.

The dipper’s breeding time advanced in response to warmer springs, a common finding in birds^2,20^. Dunn^2^ found that 79% of the studied bird species had advanced laying date with increasing spring temperatures during the last decades. In the United Kingdom, Crick and Sparks^21^ found that 27 of 45 bird species had significantly advanced breeding date over time. They^21^ found a smaller change in breeding phenology in the dipper: only −1.52 days per 1 °C in the UK compared to −4.3 days in our study. Although our study period was shorter, 34 vs 57 years, it included 13 more recent years, many of them comparatively warm. Another potential difference might be that the change in temperatures might not have been of similar magnitude. This nevertheless suggests that the response to environmental change in this species is stronger in Northern than in Western Europe^22,23^, perhaps because snow and ice conditions are more important in the north^24^.

As predicted, breeding phenology was delayed by approximately 3 days per 100 m increase in altitude. Analyses of spring phenology of terrestrial plants in Norway have shown a general delay of 2–3 days per 100 m increase in altitude^25^. Delayed egg-laying with increasing altitude has also been observed in the great tit^8,11^, suggesting that it was caused by changes in food availability and temperature. In this study we did not have such detailed data on temperature and food availability, but the strong correlation between altitude and distance from the coast (see methods), and the fact that temperature generally drops with 0.65° per 100 m increase in altitude, make a strong case for altitudinal and coastal effects being synonymous with a climatic gradient. The later breeding at higher elevations in our study may have been caused by later ice break-up at higher-elevation tributaries, since the break-up is caused by increasing spring temperatures and indicates when a territory becomes available. In essence, spring temperature measured at the regional level and local environmental conditions captured by the altitudinal especially was important in determining the timing of breeding in this study system.

Dippers in coastal areas bred earlier than inland ones, but this effect was exaggerated in years with high population density, indicating that intraspecific competition affected coastal and inland breeders differently. Competition in coastal areas advances breeding, presumably to gain competitive advantages for offspring after independence. In contrast, competition at inland areas delays breeding, indicating that competition might affect overall resource availability in the area. Presumably, territories at inland sites were marginal and when more territories were occupied, there were fewer unoccupied that could serve as extensions of occupied territories^16^.

The territory variables overall mean territory occupancy rate and overall mean territory brood size interacted with population density and contributed to explaining the variation in timing of breeding. When population density was high, dippers on often occupied territories bred earlier than those on rarely occupied territories. When population density was low, on the other hand, dippers on territories that were rarely occupied bred earlier than those on often occupied territories. For overall mean territory brood size, dippers bred earlier on territories with overall larger mean brood size when population density was low. The effect of overall mean territory brood size was evened out in high density years. This result is to some extent consistent with Sergio and Newton’s findings in the black kite^14^, where high-quality territories were occupied earlier than low-quality territories. The importance of the overall mean territory brood size indicates that not only will some territories produce larger broods, but also support earlier breeding, when population density and thus competition is low. Territories in this population are spatially fixed based on the availability of suitable nest sites in the terrain and they are used year after year. In addition, there is an obvious heterogeneity in territory occupancy rates, from being occupied only once to being occupied in all 34 years of the study. Because of the large fluctuations in population density in this population^26–28^, competition for good territories is highly variable and it is unlikely that all the variation accounted for in the analyses by territory quality only reflects that high quality pairs occupied such territories. Indeed, the results from the present study indicate that the territory quality variables included in the analyses indeed captured different aspects of territory quality. Even though overall mean territory brood size is an averaged measurement commonly used in analyses of habitat quality^14^, there is a risk that part of the correlation between reproductive output and timing of breeding is retained. The effect of overall mean territory brood size must therefore be interpreted with some caution.

Dippers breeding early raised larger broods and had more recruits than those breeding late. There was thus selection for early breeding^23,29^. However, when the fitness measure was the number of fledglings given the number of eggs laid, the optimal breeding time was moderated by spring temperature. Recruitment rate was affected by timing of breeding only, suggesting that it was breeding time per se that was most important, and not factors associated with it, like territory quality. In general, early breeding is beneficial because: (i) if food is seasonally abundant, more may be available early in the season^4,5^, (ii) offspring hatching early have more time to prepare for the winter (moult, migration or residency)^30^, (iii) early terminated breeding may improve subsequent adult survival^31^, (iv) there are more opportunities for males to become polygynous, (v) there are more opportunities for rearing second broods^32^, and (vi) more opportunities for renesting in case of failure. Note that only successful nests, i.e. nests producing fledglings, were included in the analyses (see methods for details).

The declining trend in reproductive output over the breeding season (Fig. 4) indicates a seasonal decline in feeding conditions. The dipper’s food source is mainly composed of larvae of may-, caddis- and stoneflies^33^ (Orders Ephemeroptera, Tricoptera and Plecoptera) that may spend more than a year in the water to develop. Adult stoneflies hatch during a well-defined peak, while may- and caddisfly hatch asynchronously during spring and summer. The river is, thus, unlikely to be completely depleted from food resources during the dipper’s breeding time. It is therefore not obvious that the food abundance is strongly peaked, or strongly decreasing with season, as typically found in many populations of tits^34,35^ and flycatchers^4,36,37^, which are depending on terrestrial prey. Also, as the prey is under water and therefore presumed to be less sensitive to changes in air temperature, the birds’ strong phenology response to air temperature is somewhat surprising. There might however be a potential mismatch later in the season that might hit juveniles that have reached independence^23,29^. Despite using female identity as a random effect in our analyses, there is a risk that part of the seasonal decline in reproductive success arises from differences in individual quality, where high quality females breed earlier than low quality females^23,38–40^. Consequently, it is still unclear whether the dipper responds directly to the ambient temperatures in air and water in spring, or indirectly mediated by changes in the phenology of its prey.

In conclusion, increasing spring temperatures have advanced breeding in a white-throated dipper population in southern Norway over a 34-year period. In addition, breeding time was strongly influenced by the location and the quality of the territory, where territories at low altitudes close to the fjord, particularly in high density years, were the first to hatch and thus commence breeding in spring. In low density years, birds on territories with high mean territory brood size bred earlier than those on less favourable territories, while there was no effect in high density years. Whether the abundance and the phenology of the prey proximately influenced the timing of breeding is unclear. However, early breeders produced significantly more offspring recruiting to the local breeding population than late breeders. In studies of breeding phenology, we recommend that habitat quality and altitudinal gradients are taken into account, in addition to the local weather conditions, because these might impose variation in breeding phenology not related to variation in annual spring weather. This may also be important when studying the importance of other predictor variables. Finally we found that breeding phenology may have strong effects on fitness and thus that breeding time is an important factor in a species that feeds mainly on aquatic rather than terrestrial prey.

## Materials and Methods

### Study population and data assimilation

The study population is located in the river Lyngdalselva in southernmost Norway (58° 08′–58° 40′N, 6° 56′–7° 20′E) and has been studied since 1978. Monitoring of the population is standardised (see^27^ for details). The river system of Lyngdalselva is not utilised for hydropower production or subject to other major regulations and there are no nest boxes available for breeding. It is thus a natural system for studying dipper ecology where natural nest sites are complemented by nest sites in and under bridges and other manmade structures. The study system contains a strong altitudinal gradient in addition to being located along a coastal-inland gradient, ranging from the coastal zone to 60 km inland and 700 m a.s.l. Dippers defend territories which contain one or more appropriate nest sites, which are situated so that the opening of the nest is almost always located right above fast-flowing water. Because suitable nest sites are limited and spatially segregated, there is a limited number of territories available in the river system. Within the river system we have identified a total of 158 spatially separated territories. While some of them are always occupied (34 years), others are only used a single year. It is therefore clear that some territories are preferred over others. The downstream and upstream boundaries might fluctuate slightly between years, particularly depending on whether the upstream or downstream neighbouring territories are occupied or not, but the major part of the territory will remain constant between years. The size of the study population has ranged from 20 to 117 breeding pairs over the years^27^. The number of breeding pairs with recorded phenology data is 1045, out of 2264 pairs with recorded first clutches (renesting attempts and second broods excluded). Breeding phenology was estimated as the hatching date of the first clutch of a female, according to the methods described in^41^. In summary, hatching date was estimated based on the growth trajectory of the largest nestling in each brood. This implies that the phenology of nests that did not produce young, or produced young that were not measured, could not be estimated by this method. Breeding dippers were aged and sexed according to Svensson^42^. At first encounter, breeding birds were ringed with an aluminium ring and given an individual colour code in the form of two plastic colour rings (95%). Consequently, birds did not have to be recaptured after ringing. Within the river system, the breeding outcome of almost all occupied nests was known and nearly all young were ringed.

Laying date could have been estimated based on hatching, the length of the incubation period, and the number of eggs laid, but such calculations would increase the amount of uncertainty in the estimates. In those instances where we had observed egg-laying date, there was no difference in phenology between those nests that produced young and those that failed (successful: mean = 19^th^ of April, SD = 4.5 days, N = 93; failed: mean = 22^nd^ of April, SD = 14.2 days, N = 27; two-sample t-test with Welch’s approximation for degrees of freedom: t = 0.7, df = 43, p = 0.5) and the correlation between hatching and laying date was very high (r = 0.98, df = 98, p < 0.0001).

To assess the effect of climate change, we used mean spring temperature, defined as the average temperature during Feb–Apr, provided by the Norwegian Meteorological Institute (http://om.yr.no/verdata/, see^27^ for details). The period Feb–Apr approximately encompasses the time immediately before initiation of breeding and the early stages of incubation.

Altitude and distance from the coast, indicating local climatic conditions at the location of each territory, were defined as the altitude (m a.s.l.) and the straight-line distance (m) to the river outlet of the most frequently occupied nest site within each territory. Altitude and distance from the coast were, as expected, correlated (r = 0.83, df = 156, p < 0.0001). Territory quality was measured by four different methods: (1) occupancy rate of each territory averaged over the study period, given that the territory had been visited by us, (2) the rate of successful breeding at each territory, defined as the frequency of breeding events producing at least one ringed nestling, (3) mean clutch size at each territory, defined as the mean number of eggs laid in breeding attempts producing at least one egg, and (4) mean brood size at each territory, defined as the number of ringed nestlings in breeding events producing at least one nestling.

The field work was conducted with respect for the animals’ well-being and adheres to the Guidelines for the Use of Animals in Research^43^. It complies with the laws and regulations for animals used in research in Norway. Ringing licenses were issued by the Norwegian Bird Ringing Centre.

### Statistical analyses

To describe both the trend in breeding phenology and in climate, we modelled hatching date and mean spring temperature, respectively, over time with linear mixed-effects models, using year as the random intercept. In addition, mean spring temperature was used as a predictor variable to explain the variation in breeding phenology. To avoid any spurious effects due to a temporal trend in the breeding phenology and the mean spring temperature, we included the linear trend. We also explored a possible non-linear response to spring temperature by modelling hatching date in a generalised additive mixed-effects model, and compared its fit with that of the linear model using the Bayesian Information Criterion (BIC)^44^. All statistics were performed in the program R, version 3.4.4^45^, with add-on packages ‘lme4’^46^ for linear mixed-effects models, ‘glmmADMB’ for generalized linear mixed-effects models, ‘mgcv’^47^ for generalised additive models, ‘MuMIn’^48^ for model averaging and extracting marginal and conditional R^2^ from mixed-effects models fitted with ‘lme4’, and ‘gamm4’^49^ for generalised additive mixed models. Apart from when using the ‘glmmADMB’ package which uses a slightly different method^50^ and apart from when performing likelihood ratio testing, mixed-effects models were fitted with maximum likelihood (ML) during model selection^51,52^. For Gaussian mixed models (here as well as in the further analyses), we also estimated a measure of R^2^ using package ‘MuMIn’ as the proportion of variance explained – at the level of the whole model (conditional R^2^)^53^ and at the level of fixed effects only (marginal R^2^) by means of variance partitioning^54^.

To explain the variation in the timing of breeding, we evaluated the influence of each variable on breeding phenology in an explorative setting, using model-averaging in a Gaussian linear mixed-effects model selection framework, with the function ‘dredge’ in the ‘MuMIn’ package. Models were ranked according to BIC. We also calculated Bayesian weights (BIC_w_) for each candidate model, which can be interpreted as the relative probability that a given model is the best one for the observed data, given the set of candidate models^55,56^. The variables we considered in the models were mean spring temperatures (Feb–Apr), altitude, distance from the coast, population density and the territory variables: overall mean territory occupancy rate, overall mean territory success rate, overall mean territory brood and clutch size. In addition, we included all two-way interactions with mean spring temperature and population density, respectively, because we were interested in potential interactions with these variables particularly. All models had the crossed (i.e. non-nested) random effects year, female and territory identity. Year was used as a random effect to capture annual variation not accounted for by the fixed effects, while female identity was needed due to the uneven contribution of individual females to the data. We also used territory identity as a random effect, because the study system contains a spatially fixed number of territories that do not change between years and are very different in occupancy rates, and, in addition, we need to capture territory-specific variation in the data. Only random intercept models were considered.

To address whether an early start of breeding resulted in higher fitness, we ran two parallel analyses using mixed-effects models. In the first one, the response variable was the brood size given the clutch size (the number of eggs), where the brood size more specifically is defined as the number of nestlings in a brood large enough to be ringed. In the second one, the response variable was the number of recruits given the brood size. We used generalized linear mixed-effects models with the function ‘glmmadmb’ in the ‘glmmADMB’ package^50^ and compared a range of likely models with the predictor variables hatching date, year, spring temperature, population density and altitude and the crossed random intercepts year, individual and territory identity, with a binomial error distribution, just as described above for the variation in the timing of breeding. A significant negative linear relationship between fitness and hatching date assumes that the earlier the better, which may be constrained by harsh conditions in very early spring; therefore, to control for a possible optimum in timing of breeding, we also included the quadratic effect of hatching day-of-year among the predictor variables.

## Data Availability

The data is stored at the Norwegian University of Life Sciences and is available upon request, by contacting Ole Wiggo Røstad, ole.rostad@nmbu.no. Annual averages of the data used for this article is available on Dryad.
